# Methylation Pathways and SARS-CoV-2 Lung Infiltration and Cell Membrane-Virus Fusion Are Both Subject to Epigenetics

**DOI:** 10.3389/fcimb.2020.00290

**Published:** 2020-05-26

**Authors:** Leo Pruimboom

**Affiliations:** ^1^Facultad de Enfermería y Fisioterapia Salus Infirmorum, Pontifice University of Salamanca, Madrid, Spain; ^2^PNI Europe, The Hague, Netherlands

**Keywords:** epigenetics, methylation, SARS-CoV-2, COVID-19, syncytium, cell fusion, fatality, ACE2

## Abstract

The recent pandemic SARS-CoV-2 outbreak affects all kinds of individuals worldwide. The health, social, and economic impacts of the pandemic are dramatic, and vaccines or specific treatment options are not yet available. The only approaches that we currently have available to stop the epidemic are those of classical epidemic control, such as case isolation, contact tracing and quarantine, physical distancing, and hygiene measures. It is therefore essential to find further preventive measures and possible interventions that can slow down the number of infected individuals and decrease the severity of disease when affected by SARS-CoV-2. It seems that epigenetic mechanisms are an important part of the pathophysiology and illness severity of COVID-19. These mechanisms have been identified in SARS-CoV-2 but also in other viral infections. If and when these mechanisms are confirmed, then epigenetic interventions influencing DNA methylation could be indicated as primary and/or secondary preventive options.

## Introduction

As of the day when the writing of this paper was finished, more than 4 million people worldwide had been infected, by the severe acute respiratory syndrome causing coronavirus COVID19 (SARS-CoV-2). SARS-CoV-2 is highly contagious, and the actual fatality rate is ~7% (Ferretti et al., 2020). The only approaches that we currently have available to stop the epidemic are those of classical epidemic control, such as case isolation, contact tracing and quarantine, physical distancing, and hygiene measures (Mehta et al., 2020). Coronaviruses (CoVs) infect humans and animals and cause a variety of maladies, including respiratory, enteric, renal, and neurological diseases (Corley and Ndhlovu, 2020). CoVs are classified into four different genera affecting different animals. The genera alpha-CoV and beta-CoV affect only mammals (Pinto et al., 2020) and produce mostly respiratory and gastrointestinal disorders, whereas gamma-CoV and delta-CoV infect birds and some mammals, including dolphins and white beluga whales (Rui and Sang, 2020).

Ongoing vaccine development efforts primarily focus on the coronavirus transmembrane spike (S) glycoprotein, which extends from the viral surface and mediates host cell entry (Mehta et al., 2020). The spike glycoprotein consists of two subunits, subunit S1 and subunit S2. S1 is responsible for attachment to a host molecule on the cell membrane, and S2 facilitates the fusion between the cell and virus membrane and/or between neighboring cells, producing cell–cell fusion, called a syncytium (Belouzard et al., 2012). A critical step in this crosstalk between the virus and the host cell is binding of the S1 glycoprotein to the ACE2 receptor on the surface of human cells (Hoffmann et al., 2020; Zhou P. et al., 2020) and cleavage of the spike glycoprotein by furin, a second virus receptor of COVID-19 (Abassi et al., 2020).

## Increased Expression of ACE2 and Furin Increases SARS-CoV-2 Susceptibility

Whereas early reports after the pandemic outbreak still doubted the impact of ACE2 expression on disease susceptibility (Gurwitz, 2020), more recent publications show that higher expression of ACE2 in the lungs is associated with greater disease susceptibility and severity (Leung et al., 2020). The same holds for the second identified virus receptor, furin, which is responsible for the cleavage of the S1 and S2 subunits and the consecutive endocytosis of the virus (Glinsky, 2020). The higher expression of ACE2 and furin in susceptible individuals indicates that certain epigenetic mechanisms seem to be part of the pathophysiology of SARS-CoV-2.

## Methylation and Covid-19

Epigenetics is the science of gene expression without alteration in the nucleotide sequence. Many processes influence epigenetic expression, including gene ubiquitination, histon acetylation, and, especially, DNA methylation. DNA methylation involves mostly so-called CpG islands, which are part of the promotor sequence of genes (Deaton and Bird, 2011), and the methylation pattern of CpG islands regulates the level of gene transcription (Jang et al., 2017). It has been known for years that viral infections use epigenetic mechanisms in general and especially CpG methylation to find ways to induce enterocytosis and syncytium development.

For a virus to evolve, it needs to develop a strategy to fuse itself with the cell membrane of the host and/or to induce host cell–cell fusion. Both mechanisms facilitate virus endocytosis and invasion of neighboring cells and evasion of the innate antiviral immune system (Aronson and Ferner, 2020). The type of cell formed by membrane–virus or cell–cell fusion is called a syncytium. Syncytium formation is typical for coronavirus in general, and SARS-CoV-2 is no exception (Mehta et al., 2020; Xia et al., 2020). Syncytium formation is normal in the development of the mammalian placenta, and the syncytin genes producing syncytin 1 and 2 stem from two human endogenous retroviruses (Alsaadi et al., 2019). Syncytium formation leading to the creation of giant multinucleated cells in the placenta makes this tissue impermeable and generates mother–child immune tolerance (Alsaadi et al., 2019). Syncytin genes are hypomethylated and therefore functionally active in mammalian placenta, whereas they are hypermethylated, and thus silenced, in other tissues, were syncytium formation may cause various diseases, including schizophrenia, multiple sclerosis, and diabetes type 1 (Dupressoir et al., 2012). CpG methylation of syncytin genes in non-placental tissues is obligatory for the prevention of expression of syncytium-forming proteins (Matoušková et al., 2006). Several viruses use the human syncytin genes to fuse themselves with the cell membrane of the host and/or to induce cell–cell fusion in the infiltrated tissues (Levet et al., 2019). Good examples of how viruses can use epigenetic mechanisms to fuse themselves with host cells are given by the way the Epstein-Barr virus and the cytomegalovirus can affect human health. Both viruses are able to demethylate the host syncytin 1 and 2 genes, increasing gene transcription and causing syncytium formation in tissues where those genes are normally hypermethylated and silenced (Esteki-Zadeh et al., 2012; Niller et al., 2014). This process can cause diseases such as multiple sclerosis and even amyotrophic lateral sclerosis (Küry et al., 2018). Syncytium formation by SARS-CoV-2 is many times faster than in the 2002 SARS-CoV, and syncytium formation is highly responsible for the virulence factor and induction of a cytokine storm of any virus in general and SARS-CoV-2 especially (Matsuyama et al., 2020; Xia et al., 2020).

## Evidence of Epigenetic Mechanisms in SARS-CoV-2 Susceptibility and Disease Severity (Figure 1)

Two recent publications (Corley and Ndhlovu, 2020; Pinto et al., 2020) identified the importance of the methylation pattern of the gene encoding for angiotensin-converting enzyme 2, known to be the most important virus receptor on host lung epithelial cells for SARS-CoV-2 (Zill et al., 2012; Rui and Sang, 2020). It has been shown that the production rate of the ACE2 enzyme by its gene is under epigenetic control (Zill et al., 2012). The results by Corley and Ndhlovu (2020) reveal that the ACE2 gene activity, based on the methylation pattern of the several promotor CpG isles, is associated with age and gender. ACE2 is present in multiple human tissues and organs, including the lung, the gut, the liver, the pancreas, the brain, and blood. The methylation rate in lung epithelial cells was the lowest compared with all the other tissues, which suggests that lung tissue has the highest transcription and expression rate of ACE2 (Corley and Ndhlovu, 2020). At the same time, it was evidenced that the ACE2 gene in neurons and leukocytes is hypermethylated and that the protein seems not to be expressed. Age correlates in this study with hypomethylation of the ACE2 gene in lung tissue, which could provide a partial explanation for aging as a risk factor for SARS-CoV-2 fatality, whereas male gender shows a trend in hypomethylation. The results of another study (Pinto et al., 2020) add evidence to the findings of the study of Corley. In this study, 700 lung transcriptome samples of patients with comorbidities and suffering from severe SARS-CoV-2 were analyzed, and it was found that ACE2 was highly expressed in these patients compared to control individuals (Pinto et al., 2020).

**Figure 1:**
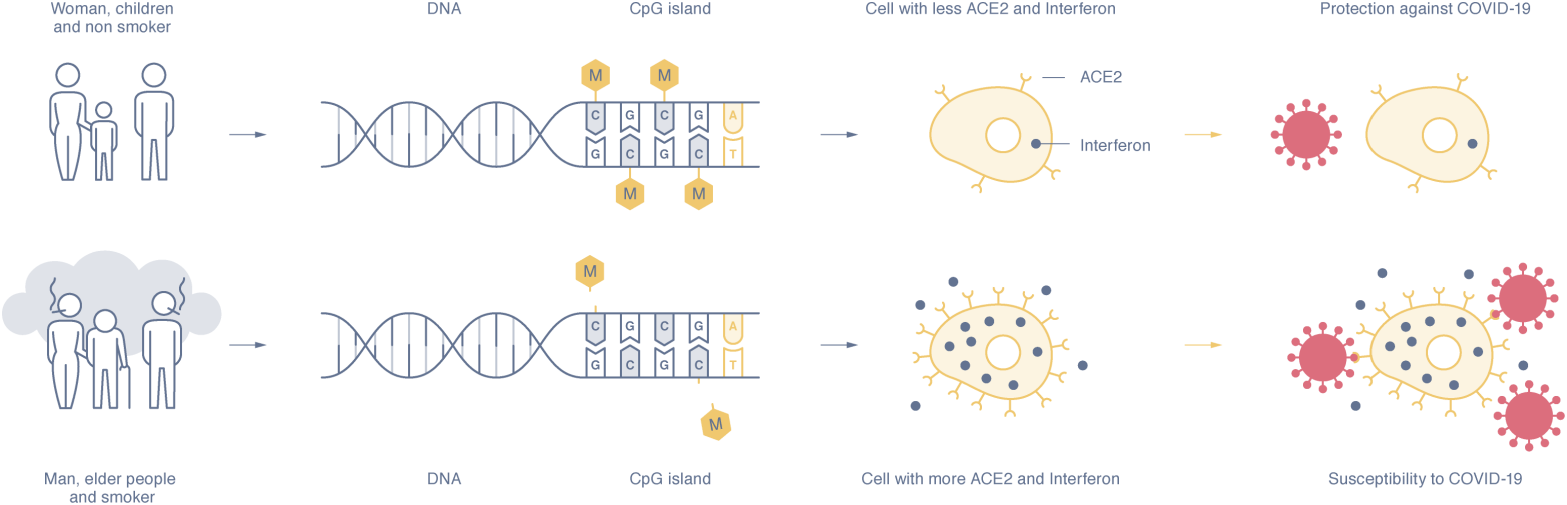
The expression of the ACE2 and interferon gene depends on the methylation rate of the CpG islands in the DNA promotor sequence. Susceptible individuals, mostly men, the elderly, and smokers, show a hypomethylation pattern of the ACE2 and interferon genes (lower part), whereas women, children, and non-smokers show DNA hypermethylation and lower expression of ACE2 and interferon proteins (upper part). The higher presence of ACE2 on epithelial cells and interferon makes people more susceptible to SARS-CoV-2 infection and increases disease severity, whereas a low presence of ACE2 and interferon seems to offer disease protection.

The summarized results from research into the association of the methylation pattern of ACE2, gender, and age with SARS-CoV-2 susceptibility and disease severity explain the still preliminary epidemiological data indicating that age and male gender are risks factors for the development of more severe disease and fatality (Jin et al., 2020; Ruan, 2020). Age was shown to be strongly associated with mortality (Ruan, 2020), whereas the male fatality rate in a population of 43 patients (male *n* = 22, female *n* = 21) was 70%, independent of age or susceptibility (Jin et al., 2020). The latter could be explained by the much higher rate of smoking in male than in female individuals in countries such as China, Spain, and Italy, where the disease susceptibility and mortality of males is extremely high (Brake et al., 2020). Cai (2020) recently reported higher ACE2 gene expression in smoker samples compared to never-smokers, and these data were confirmed by a study of Leung (Leung et al., 2020), again highlighting the epigenetic regulation of ACE2 as essential for SARS-CoV-2. Next to smoking as a risk factor for SARS-CoV-2, testosterone also seems responsible for higher expression of ACE2 and furin in men (Glinsky, 2020).

Epigenetic regulation and increased expression of ACE 2 in both oral space and lung tissue may explain why older people are more sensitive to the development of symptomatic SARS-CoV-2 than younger people and especially children (Pinto et al., 2020). This is consistent with the process of epigenetic aging, which has been shown to cause certain genes to gradually become more active during the aging process and others to become more inactive (Jones et al., 2015). One of the more active genes is ACE 2, and this makes older people more susceptible to viral infections and therefore also to COVID-19. The opposite applies to children. In children, the ACE 2 gene in the lungs, oral tissues, and other organs is normally hypermethylated and therefore virtually silenced (Holmes et al., 2019).

The abovementioned data support the notion that epigenetic mechanisms are involved in multiple mechanisms with which SARS-CoV-2 infects the human host. This could also mean that certain subgroups of patients with known epigenetic characteristics are more susceptible to SARS-CoV-2. One such subgroup could be those patients suffering from systemic lupus erythematous (SLE). SLE patients are possibly more prone to developing SARS-CoV-2 symptoms, not so much because of a compromised immune system but because of strong overexpression of the lung ACE2 protein and the related hypomethylation of its gene, together with a significant level of demethylation of interferon genes (Sawalha et al., 2020). Higher expression of interferon genes has been related to the disease development of severe SLE, characterized by a cytokine storm (Walden et al., 2019), and a cytokine storm is characteristic of SARS-CoV-2 (Mehta et al., 2020). All of these molecular details relating to SLE fulfill the conditions of increased COVID-19 susceptibility and increased disease severity. A recent report confirms the possible increased susceptibility to and disease severity from SARS-CoV-2 in patients with SLE, and its authors also note the need of more studies because of the fact that patients with SLE have a high prevalence of comorbidities, such as lung diseases, chronic kidney disease, and obesity (Mathian et al., 2020).

## Epi-Drugs as Possible Treatment/Vaccine Options for SARS-CoV-2

Epigenetics as a science is still in its early development. It is nevertheless possible to influence the epigenetic regulation of multiple genes with natural interventions. The use of vitamin D and quercetin could be interesting for ameliorating SARS-CoV-2 severity by inhibiting the expression of ACE2 and furin, although the study suggesting this intervention is based on *in vitro* data and is still not peer-reviewed (Glinsky, 2020). Nevertheless, Ilie and Smith (2019) found that the average vitamin D level in European countries correlates negatively with the mortality rate of SARS-CoV-2, and this supports the still somewhat preliminary recommendation of using vitamin D as a preventive intervention for SARS-CoV-2. Other candidates for epigenetic silencing of ACE2 and interferon genes are curcumin, deferasirox, and 8-hydroxyquinolones (8HQ) (Sfera et al., 2018). Curcumin is a potent activator of DNA methyltransferases in viable clinical doses (Hassan et al., 2019). Another so-called epi-drug with proven methylation capacity is sulforaphane (from broccoli, Kaufman-Szymczyk et al., 2015). All of these substances are over-the-counter natural medicines and could help to attenuate disease severity and susceptibility. Curcumin is especially interesting because of its ferritin-lowering effects (Sfera et al., 2017), given that increased ferritin values in patients suffering from severe SARS-CoV-2 worsen the outcome significantly (Zhou F. et al., 2020).

## Discussion

SARS-CoV-2 has caused a devastating pandemic worldwide, with huge consequences not only for health but for economies. Vaccine development can take months to years, and it is therefore essential to find ways to decrease virus infection and disease severity. Epigenetic pathways seem crucial for the pathophysiology of COVID-19, and all the essential host substances acting as virus receptors show higher expression in susceptible individuals, including males, smokers, and elderly people. Stimulation of specific DNA methylation of ACE2, furin, and interferon genes could help to attenuate contamination susceptibility and disease severity, and vitamin D and curcumin should be considered as epi-drugs and regulators of DNA expression. It should be obvious that the epigenetic data associated with SARS-CoV-2 infection and disease severity have to be confirmed by more epidemiologic studies before any thorough recommendations about the use of epi-drugs can be made. Nevertheless, curcuma supplementation, ceasing smoking, and the use of safe doses of vitamin D will definitely not cause any harm and will possibly help to ameliorate SARS-CoV-2 infection and disease severity in susceptible individuals.

## Author Contributions

The author confirms being the sole contributor of this work and has approved it for publication.

## Conflict of Interest

LP was employed by PNI Europe.
